# Supplementary material for: Hallmarks of glycogene expression and glycosylation pathways in squamous and adenocarcinoma cervical cancer
Source: PeerJ. 2021 Aug 31;9:e12081. doi: 10.7717/peerj.12081 (PMC8415283; doi:10.7717/peerj.12081)
Supplement: Supplemental Information 3 [file peerj-09-12081-s003.docx]

**MIAME**

**Part 1 Experiment description**

-Normal vs cervical cancer tissues comparison. Cervical cancer tissues correspond to patients without previous treatment.

-Experimental variables: not applied

N count- in the normal group were included 15 samples and in the cervical cancer group were included 23 samples.

**Part 2 Array design**

Human 50-mer oligo library set “A” from MWGBiotech Oligo Sets (http:/www.mwgbiotech.com) was used. It contains 10,000 gene specific oligonucleotide probes representing the best-annotated genes from human. Oligo library was resuspended to 50µM in Micro Spotting solution (ArrayIt Brand Products.). SuperAmine coated slides 25x75 mm (TeleChem International INC) were printed in duplicate, and fixed at 80°C for 4 hours. For pre-hybridization the slides were re-hydrated with water vapor at 60°C, and fixed with two cycles of UV light (1200J). After boiling for two minutes at 92°C, slides were washed with 95% ethanol for one minute and prehybridzed in 5X SSC, 0.1% SDS and 1% BSA for one hour at 42°C. The slides were washed and dried for further hybridization.

**Part 3 Samples**

Probe preparation and hybridization to arrays. 10 µg of total RNA were used for cDNA synthesis incorporating dUTP-Alexa555 or dUTP-Alexa647 employing the Firs-Strand cDNA labeling kit (Invitrogen). Incorporation of fluorophore was analyzed by using the absorbance at 555 nm for Alexa555 and 650 nm for Alexa647.

Normal cervix samples were obtained from women undergoing a hysterectomy for uterine myomatosis. Squamous CC samples were obtained from patients with no previous treatment. A total of 23 CC samples and 15 biopsies of normal cervical tissue were included.

Normal cervix tissues and CC samples were collected and maintained in RNAlater Solution (Qiagen, Hilden, Germany) at -80°C. Total RNA was extracted using the RNeasy Plus system (Qiagen, Hilden, Germany) following the manufacturer’s protocol. RNA samples were pooled into two groups (normal and CC).

**Part 4 Hybridizations**

Equal quantities of labeled cDNA were hybridized using hybridization solution UniHyb (TeleChem International INC). The arrays were incubated for 14 h at 42°C, and then washed tree times with 1X SCC, 0.05 % SDS at room temperature.

**Part 5 Measurements**

Acquisition and quantification of array images was performed in GenePix 4100A with its accompanying software GenePix from Molecular Devices. All images were captured using 10µm resolution. For each spot the Alexa555 and Alexa647 density mean value and background mean value were calculated with software ArrayPro Analyzer from Media Cibernetics.

Microarray data analysis was performed with free software genArise, developed in the Computing Unit of Cellular Physiology Institute of UNAM (http://www.ifc.unam.mx/genarise/). GenArise carry out a number of transformations: background correction, lowess normalization, intensity filter, replicates analysis and selecting differentially expressed genes. The goal of genArise is to identify which of the genes show good evidence of being differentially expressed. The software identifies differential expressed genes by calculating an intensity-dependent z-score. Using a sliding window algorithm to calculate the mean and standard deviation within a window surrounding each data point and define a z-score where z measures the number of standard deviations a data point is from the mean.

zi = (Ri – mean(R)) / sd(R)

Where zi is the z-score for each element, Ri is the log-ratio for each element, and sd(R) is the standard deviation of the log-ratio. With this criterion, the elements with a z-score > 2 standard deviations would be the significantly differentially expressed genes.

**Part 6 Normalization controls**

Here we tested whether cervical cancer displays alteration in the glycogene expression compared with the normal cervix. The overall design consisted of a two-condition experiment: normal cervix tissue and cervical cancer tissue. Microarray data analysis was performed with free software genArise and a lowess normalization was performed. Changes in gene expression in cervical cancer were relative to the normal cervix. Genes with a Z score ≥ 2 or ≤ -2 were considered with altered expression. Results indicate that in comparison with the normal cervix, cervical cancer displays upregulation in genes associated with the GPI-anchored biosynthesis pathway and downregulation in glycogenes related to the synthesis of chondroitin and dermatan sulfate.
