# Supplementary figures and images for: Hallmarks of glycogene expression and glycosylation pathways in squamous and adenocarcinoma cervical cancer
Source: PeerJ. 2021 Aug 31;9:e12081. doi: 10.7717/peerj.12081 (PMC8415283; doi:10.7717/peerj.12081)

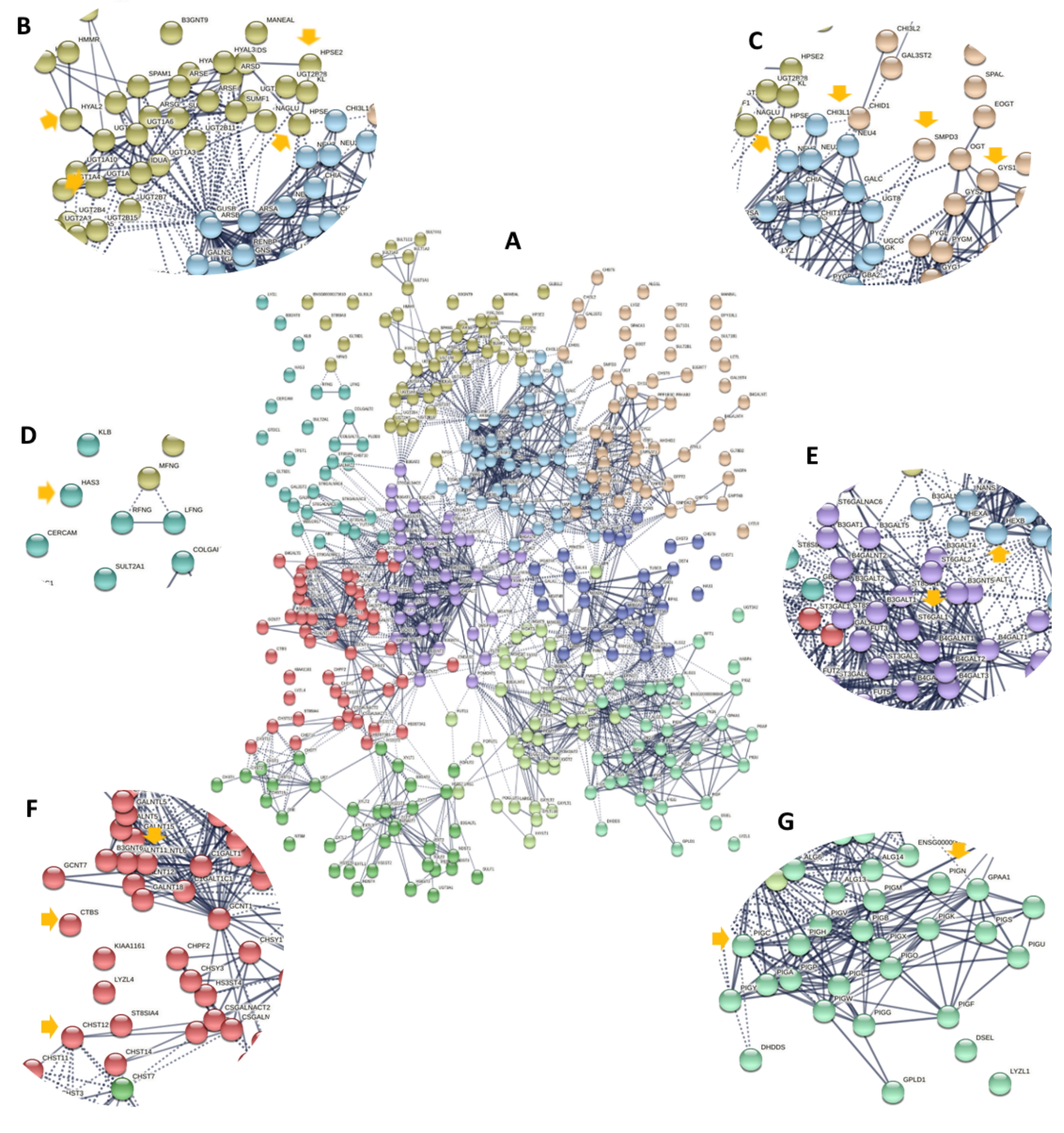

Supplement: Supplemental Information 5 — (A) Predicted protein-protein interaction among a total of 401 glycogenes grouped in ten clusters showing an intricated connection; each color indicates a cluster. (B) The downregulated glycogenes HYAL2, HPSE, UGT2B4, and UGT2B28 are grouped in the green cluster. (C) SMPD3 and GYS1 are in the pink cluster, while CHI3L1 is located in the blue cluster near the green cluster. (D) HAS3 is localized in the soft blue cluster. (E) ST6GAL1 is mapped in the violet cluster that displays a network. (F) CHST12, CTBS, and GALNT11 are grouped in the red cluster. (G) The upregulated glycogenes PIGC and PIGN are localized in the turquoise cluster. (PPI p-value < 1.0e − 16, interaction score 0.7, kmeans clustering, STRING version 11.0). [file peerj-09-12081-s005.jpg]

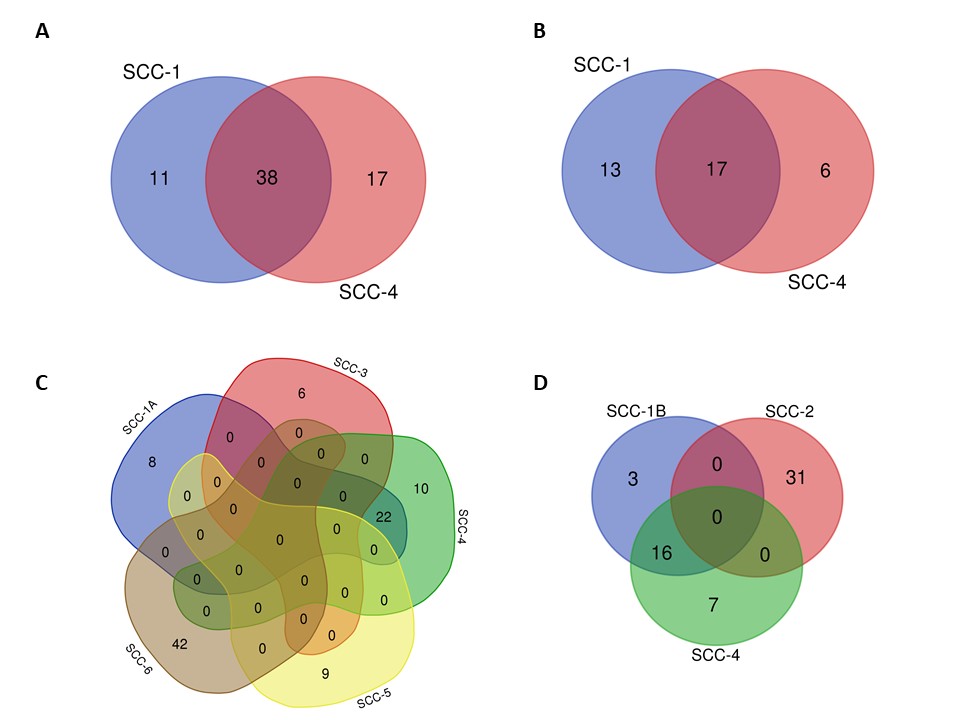

Supplement: Supplemental Information 6 — (A) SCC-1 and SCC-4 contained 38 glycogenes in common and comprised the same 17 patients’ samples (B). (C) Differentially expressed glycogenes in SCC-1, SCC-3, SCC-4, SCC-5, and SCC-6; respectively: eight, six, ten, nine, and 42 glycogenes in each case. (D) Differentially glycogenes with low expression in SCC-1, SCC-2, and SCC-4; respectively: three, 31, and six genes. SCC: squamous carcinoma cluster. [file peerj-09-12081-s006.jpg]
